# Supplementary material for: Content-rich biological network constructed by mining PubMed abstracts
Source: BMC Bioinformatics. 2004 Oct 8;5:147. doi: 10.1186/1471-2105-5-147 (PMC528731; doi:10.1186/1471-2105-5-147)
Supplement: Additional File 2 — The original results of the above study (non-essential files are deleted to keep the file size under the limit set by BMC bioinformatics). [file 1471-2105-5-147-S2.bz2 › chilibotAdditionalFile2/dip10/721ID11799122E18058/html/ENVZ_OMPR.html]

 


 **ENVZ** and **OMPR** 
  
Found 178 abstracts in PubMed, retrieved 10.  
 

 What does Google say? 
 PDF only 
| .edu only 

---

**Interactive relationship** (e.g. stimulation, inhibition, etc)

**Neutral relationship**- Interaction of  **EnvZ** , a sensory histidine kinase, with phosphorylated  **OmpR** , the cognate response regulator.  Ref: 12453215 Mol Microbiol, 2002
- The osmosensor,  **EnvZ** , has dual enzymatic functions with  **OmpR**  kinase and  **OmpR**  P phosphatase.  Ref: 12453214 Mol Microbiol, 2002
- We demonstrated that  **OmpR**  P is able to interact with EnvZc with a similar affinity to  **OmpR**  and serves as an effective substrate for the  **EnvZ**  phosphatase.  Ref: 12453215 Mol Microbiol, 2002
- **OmpR**  P bound to EnvZc was readily released upon the addition of  **OmpR** , indicating that  **OmpR**  and  **OmpR**  P can compete for the binding to  **EnvZ** .  Ref: 12453214 Mol Microbiol, 2002
- The model predicts that when  **EnvZ**  is much less abundant than  **OmpR** , as is the case in E. coli, the steady state level of phosphorylated  **OmpR**   **OmpR**  P is insensitive to variations in the concentration of  **EnvZ** .  Ref: 12522261 Proc Natl Acad Sci U S A, 2003
- **EnvZ**  is a sensory histidine kinase in Escherichia coli to regulate the phosphorylation of  **OmpR** , its cognate response regulator, required for the expression of genes for outer membrane porin proteins.  Ref: 12453215 Mol Microbiol, 2002

**Non-interactive relationship** (e.g. studied together, co-existance, homology, etc.)

- Formation of the stoichiometric complex of  **EnvZ** , a histidine kinase, with its response regulator,  **OmpR** .  Ref: 12453214 Mol Microbiol, 2002
- The  **EnvZ**   **OmpR**  system in Escherichia coli, which regulates the expression of the porins OmpF and OmpC, is one of the simplest and best characterized examples of two component signaling.  Ref: 12522261 Proc Natl Acad Sci U S A, 2003
- The operon flhDC is controlled by numerous regulators including H NS, CRP,  **EnvZ**   **OmpR** , QseBC and LrhA.  Ref: 12864862 Mol Microbiol, 2003
- Using these strains we have shown that, consistent with the predictions of our model, the transcription of ompC and ompF is indeed robust or insensitive to a wide range of expression levels of both  **EnvZ**  and  **OmpR** .  Ref: 12522261 Proc Natl Acad Sci U S A, 2003
- **EnvZ** , a histidine kinase, and its cognate response regulator  **OmpR**  of Escherichia coli are responsible for adaptation to external osmotic changes by regulating the levels of the outer membrane porin proteins, OmpF and OmpC.  Ref: 12453214 Mol Microbiol, 2002
- The  **EnvZ**   **OmpR**  histidyl aspartyl phosphorelay HAP system in Escherichia coli regulates the expression of ompF and ompC, the major outer membrane porin genes, in response to environmental osmolarity changes.  Ref: 12767831 J Mol Biol, 2003
- Partial sequence analysis of a large native plasmid approximately 120 kb, designated pA5H5, from a highly virulent Australian isolate revealed a putative two component regulatory system with inferred identity to the  **OmpR**  family of two component transcriptional regulatory proteins and  **EnvZ**  sensor kinases.  Ref: 12398213 Int J Med Microbiol, 2002
- These findings support the hypothesis that osmotic signals regulate the level of the cellular concentration of  **OmpR**  P by modulating the ratio of kinase to phosphatase activity of the bifunctional enzymatic activities of  **EnvZ** .  Ref: 12453215 Mol Microbiol, 2002
- To test these predictions, we have perturbed the porin regulatory circuit in E. coli by varying the expression levels of  **EnvZ**  and  **OmpR** .  Ref: 12522261 Proc Natl Acad Sci U S A, 2003
- The phosphoryl group of H6 HepK is transferred rapidly and efficiently to both H6DevR and H6DevRA but not to His tagged  **OmpR** , whose cognate sensor kinase is  **EnvZ** .  Ref: 12637541 J Biol Chem, 2003
- Here, we demonstrate that the cytoplasmic kinase domain of  **EnvZ**  EnvZc and  **OmpR**  are able to form a 1 1 complex detected by native PAGE.  Ref: 12453214 Mol Microbiol, 2002
- We have analyzed a mathematical model of the  **EnvZ**  mediated cycle of  **OmpR**  phosphorylation and dephosphorylation.  Ref: 12522261 Proc Natl Acad Sci U S A, 2003
- **EnvZ**  and  **OmpR**  are a transmembrane sensor and its cognate response regulator, respectively, regulating the transcription of porin genes in response to medium osmolarity in Escherichia coli.  Ref: 12754242 J Bacteriol, 2003
- Here, we re examined the recent paper Mattison and Kenney, in which the authors reported that phosphorylated  **OmpR**   **OmpR**  P is unable to bind to  **EnvZ** , thus casting doubts on the role of the  **EnvZ**  phosphatase activity in vivo.  Ref: 12453215 Mol Microbiol, 2002
- In alpha proteobacteria, HPRK is located downstream of genes encoding a two component system of the  **EnvZ**   **OmpR**  family.  Ref: 12867744 J Mol Microbiol Biotechnol, 2003
- A variety of genes hilC, hilD, fis, sirA barA, csrAB, phoB, fadD,  **envZ**   **ompR** , fliZ, hilE, ams, lon, pag, and hha have been identified that exert positive or negative effects on hilA expression, although the mechanisms by which these gene products function remain relatively unclear.  Ref: 12595445 Infect Immun, 2003
